# miR-1297 is frequently downmodulated in flat epithelial atypia of the breast and promotes mammary neoplastic transformation *via* EphrinA2 regulation

**DOI:** 10.1186/s13046-025-03354-2

**Published:** 2025-03-14

**Authors:** Giorgia Scafetta, Gian Luca Rampioni Vinciguerra, Simona Giglio, Omar Faruq, Roberto Cirombella, Ilenia Segatto, Francesca Citron, Maria Chiara Mattevi, Elisabetta Di Renzi, Luciano Cascione, Pierluigi Gasparini, Barbara Belletti, Gustavo Baldassarre, Andrea Sacconi, Giovanni Blandino, Andrea Vecchione

**Affiliations:** 1 Department of Clinical and Molecular Medicine, Faculty of Medicine and Psychology, Sant’Andrea Hospital, University of Rome “Sapienza”, 00189 Rome, Italy; 2 Unit of Molecular Oncology, Centro di Riferimento Oncologico di Aviano (CRO), IRCCS, National Cancer Institute, 33081 Aviano, Italy; 3 Institute of Oncology Research, Faculty of Biomedical Sciences, USI, Bellinzona, Switzerland; 4 Department of Cancer Biology and Genetics and Comprehensive Cancer Center, The Ohio State University, Columbus, OH 43210 USA; 5 Clinical Trial Center, Biostatistics and Bioinformatics Unit, IRCCS Regina Elena National Cancer Institute, Rome, Italy; 6 Translational Oncology Research Unit, IRCCS Regina Elena National Cancer Institute, Rome, Italy

**Keywords:** Breast cancer, Flat epithelial atypia, Mammary transformation, miR-1297, EphA2

## Abstract

Breast cancer ranks as the most prevalent form of cancer globally. Currently, advanced screening methods have significantly improved early detection rates. These achievements have led to more non-invasive cancer diagnoses and underscored the clinical relevance of precursor lesions like flat epithelial atypia (FEA), a histological condition characterized by mild atypical changes in the normal epithelium lining the mammary ducts. Despite the increasing detection of FEA in mammary biopsy, our understanding of the biological behavior of this entity remains limited and, as a consequence, the clinical management of patients is still being debated. Evidence from the literature indicates that dysregulation of microRNAs contributes to all stages of breast cancer progression, potentially serving as valuable markers of disease evolution. In this study, through a comparison of the microRNA profiles of normal mammary epithelium, FEA, and non-invasive breast cancer in three cohorts of patients, we identified downregulation of miR-1297 as a common feature in both FEA and non-invasive breast cancer compared to the normal counterpart. Mechanistically, overexpression of miR-1297 inhibits the growth of breast cancer cells by targeting the oncogenic receptor tyrosine kinase EphrinA2. In contrast, downregulation of miR-1297 increases proliferation and alters the morphology of normal mammary epithelial cells in a three-dimensional context. These findings pinpoint the downregulation of miR-1297 as an early event in mammary transformation and suggest its potential role as a driver of progression in FEA, harboring the capacity to evolve into malignancy.

**Supplementary Information:**

The online version contains supplementary material available at 10.1186/s13046-025-03354-2.

## Introduction

Breast cancer (BC) is the most common cancer worldwide and the second-leading cause of cancer-related death among women [1, 2]. To date, the development and implementation of effective screening campaigns have drastically changed the natural evolution of BC, notably reducing advanced disease occurrences and overall mortality rates [1, 3]. On the other hand, those improvements have also resulted in the overdiagnosis of non-invasive breast lesions in biopsy, whose biological behavior remains subject to debate [1]. Flat epithelial atypia (FEA) is considered the earliest histologically recognizable, neoplastic alteration of the breast [4]. Morphologically, FEA is characterized by the replacement of the native epithelium of mammary ducts with atypical, monomorphic columnar cells arranged in a “flat” pattern of growth [4, 5]. FEA represents a presumably neoplastic lesion [4, 5] and, despite the detection frequency is increasing over time [6, 7], its clinical management has remained controversial. Historically, FEA detected on needle core biopsy has typically led to surgical excision. However, this approach has raised doubts regarding potential overtreatment [8], given the low rate at which FEA detected on biopsy is associated with malignancy in the subsequent surgical specimens [9]. Current guidelines advise against open excision for biopsy-proven FEA, advocating for a more conservative approach centered around vacuum-assisted excision [10].

Nevertheless, FEA is still regarded as a nonobligate precursor of breast cancer with frequent genetic alterations [11] and a prolonged progression timeline [4]; therefore, active surveillance of patients is firmly recommended [10]. To date, few studies have investigated the molecular features of FEA [11], and the mechanisms underlying the progression to BC remain largely unclarified. Furthermore, no clinical or pathological criteria are available to stratify the malignant potential of FEA cases and provide guidance for patient management decisions [9].

MicroRNAs (miRNAs) are a class of short non-coding RNAs, approximately 20 nucleotides in length, that regulate the gene expression at the post-transcriptional level, thereby playing a complex biological role in several normal and pathological processes [12, 13]. The miRNAome is frequently dysregulated in human malignancies, functionally contributing to the tumor onset and progression [14–17]. From a translational perspective, it is largely established that specific microRNAs may act as potential biomarkers to predict the clinical behavior of human malignancies, the survival of patients, the response to therapies and the possibility of recurrences [18–20]. Aberrant expression of microRNAs has been identified at every stage of BC development, including the pre-invasive stage of carcinogenesis [21]. In this regard, investigating the role of microRNAs in non-invasive ductal carcinoma *in situ* (DCIS), our group has recently demonstrated that miR-223 downmodulation is an early event during mammary tumor onset and contributes to the progression from non-invasive to invasive BC [22]. However, no microRNA profiling has been conducted in FEA lesions and the possible role of microRNAs in the very early stages of mammary transformation remains largely elusive.

In this study, through conducting microRNA expression analysis on FEA, DCIS, and normal mammary epithelium (NME), we identified miR-1297 downregulation as a critical event in the early transformation of mammary epithelium and highlighted the role of its target EphA2 in the establishment of BC.

## Results

### miR-1297 downmodulation is a common feature of FEA and DCIS

Flat epithelial atypia (FEA) is considered the earliest histologically recognizable, neoplastic alteration of the breast [11]. Despite several studies that have explored the clinical significance of FEA and its association with BC [6–9], the biological mechanisms underlying the onset and progression of FEA and the possible role of microRNAs in these processes are largely unexplored.

**Fig 1.**
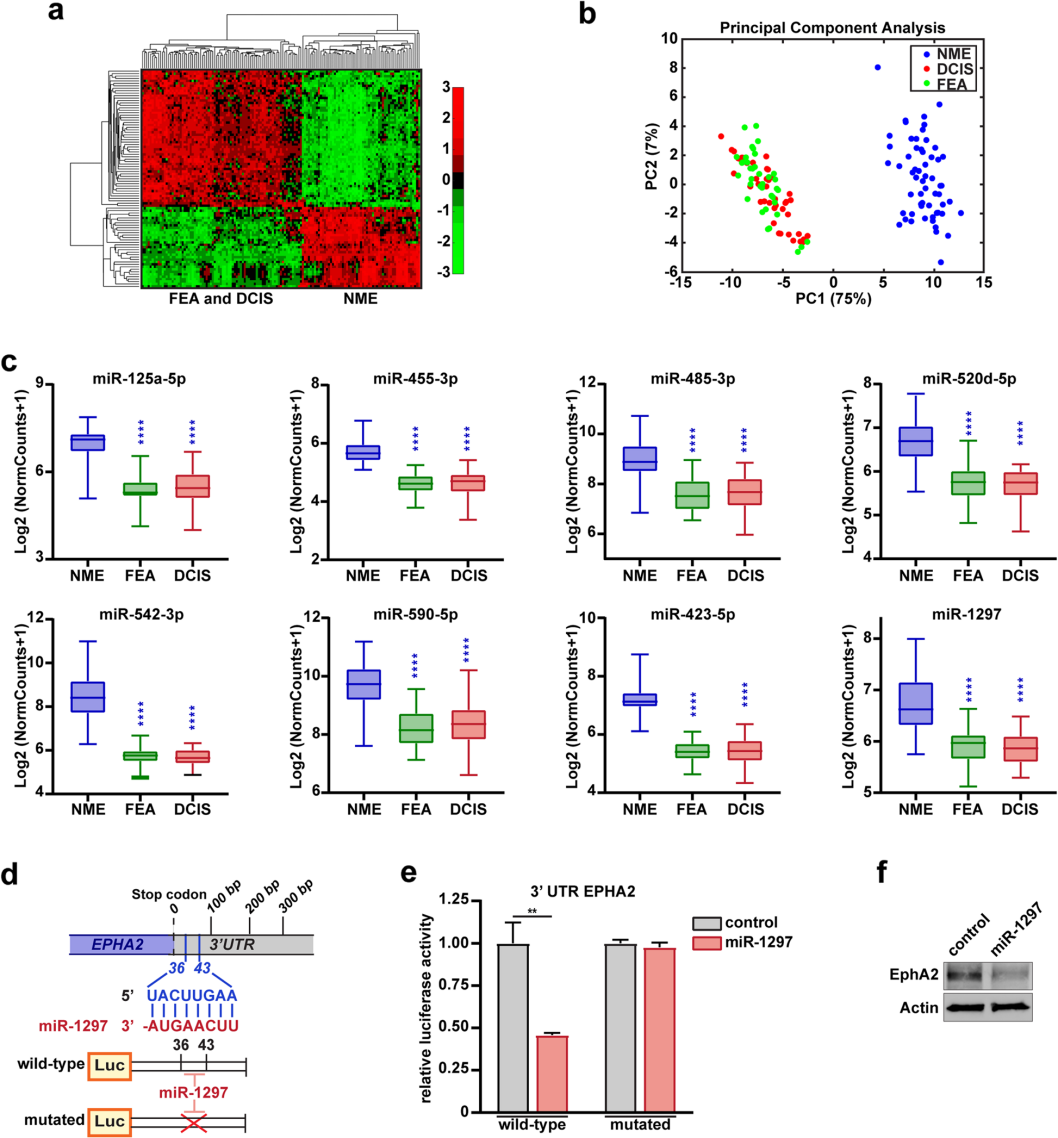
miR-1297 downmodulation is a common feature of FEA and DCIS. **a**, Heat map of deregulated microRNAs in flat epithelial atypia (FEA, n=38) and non-invasive ductal carcinoma in situ (DCIS, n=42) compared to normal mammary epithelium samples (NME, n=59) collected from patients with breast. **b,** Principal Component Analysis of 89 miRNAs significantly modulated between FEA and DCIS versus NME samples. The first component accounts for most of the variance that distinguishes NME samples from FEA and DCIS samples. **c,** Box plots illustrating the normalized expression of the most interacting miRNAs downregulated in FEA and DCIS compared to NME. Statistical significance was assessed using the Mann–Whitney test. **d,** Schematic representation of miR-1297 binding site on EPHA2 3'UTR and its deletion. **e,** Histogram representing the normalized luciferase activity of pMir Target vector with EPHA2 WT 3’UTR insert and EPHA2 mutated 3’UTR. Luciferase activity was measured after 48 h post-transfection in MCF7 cells. Data represent the mean ±SD from three independent experiments and statistical significance was evaluated by unpaired t-test. **f**, Western blot analysis evaluating the expression of EphA2 in lysates from control and miR-1297 transfected MCF7 cells, as outlined in **e**. Actin was used as the loading control. In **c** and **e**, Asterisks indicate significant differences. ** *p*-value <0.01; **** *p*-value <0.0001.

To this aim, we performed an expression analysis in an explorative cohort of microdissected BC samples collected from our Institution, comparing microRNAs levels in FEA (38 samples), DCIS (42 samples) and in the normal mammary epithelium (NME, 59 samples). Among the about 800 detected microRNAs, we found that a signature of 89 microRNAs could distinguish NME from FEA and DCIS samples, as revealed by principal component analysis (Figure 1a, b, and Supplementary Table 1).

Literature data show that microRNA downmodulation represents a common trait in malignant transformation, particularly in BC [22–24]. Thus, we decided to focus on the 33 downregulated microRNAs, wondering whether those molecules could exert a role in the malignant progression of FEA. Conducting a network analysis using OmicsNet software [25] (Supplementary Figure 1a), we identified a signature comprising 8 microRNAs (miR-125a-5p, miR-455-3p, miR-485-3p, miR-520d-5p, miR542-3p, miR-590-5p, miR-423-5p, and miR-1297) that were notably downmodulated in FEA/DCIS compared to NME (Figure 1c). These microRNAs shared validated targets that belong to essential pathways associated with breast carcinogenesis (Supplementary Figure 1b and Supplementary Table 2, 3), further supporting their possible involvement in the evolution of BC.

Our attention was drawn to miR-1297, a microRNA that plays a critical role in human diseases, including cancer, where its function varies in a context-dependent manner [26]. In BC, miR-1297 has been reported to exhibit an ambivalent role [21, 26], with evidence highlighting its downregulation as a characteristic feature of tumor tissues [27]. Furthermore, its expression has been positively correlated with longer survival in triple-negative BC patients [28], further supporting its potential role as a tumor suppressor.

By analyzing the predicted targets of miR-1297 using TargetScan software [29], we identified Ephrin A2 (EphA2) as a potential candidate. EphA2 is a receptor tyrosine kinase with oncogenic functions frequently reported to be dysregulated in BC, contributing to the transformation of mammary epithelial cells [30–32]. Since EPHA2 harbors a conserved seed region in its 3’UTR, we investigated whether miR-1297 downmodulation could play a role in our model *via* EphA2 upregulation.

We thus cloned the 3′UTR miR-1297-binding sequence of EPHA2 into the pMir Target vector (Figure 1d) and used this plasmid to assess the binding miR-1297 and its activity on the EPHA2 3’UTR by performing luciferase assays in MCF7 BC cells. We observed a significant decrease in luciferase activity upon miR-1297 expression, which was rescued when the miR-1297 seed-binding site was mutated (Figure 1e). Furthermore, miR-1297 expression markedly reduced EphA2 protein levels compared to the control (Figure 1f).

These findings indicate that miR-1297 downmodulation associates FEA with DCIS and distinguishes these early neoplastic lesions from NME. Interestingly, miR-1297 directly targets EphA2, a pivotal player in mammary transformation.

### FEA is associated with miR-1297 downmodulation and EphA2 overexpression in an independent cohort of patients

**Fig 2.**
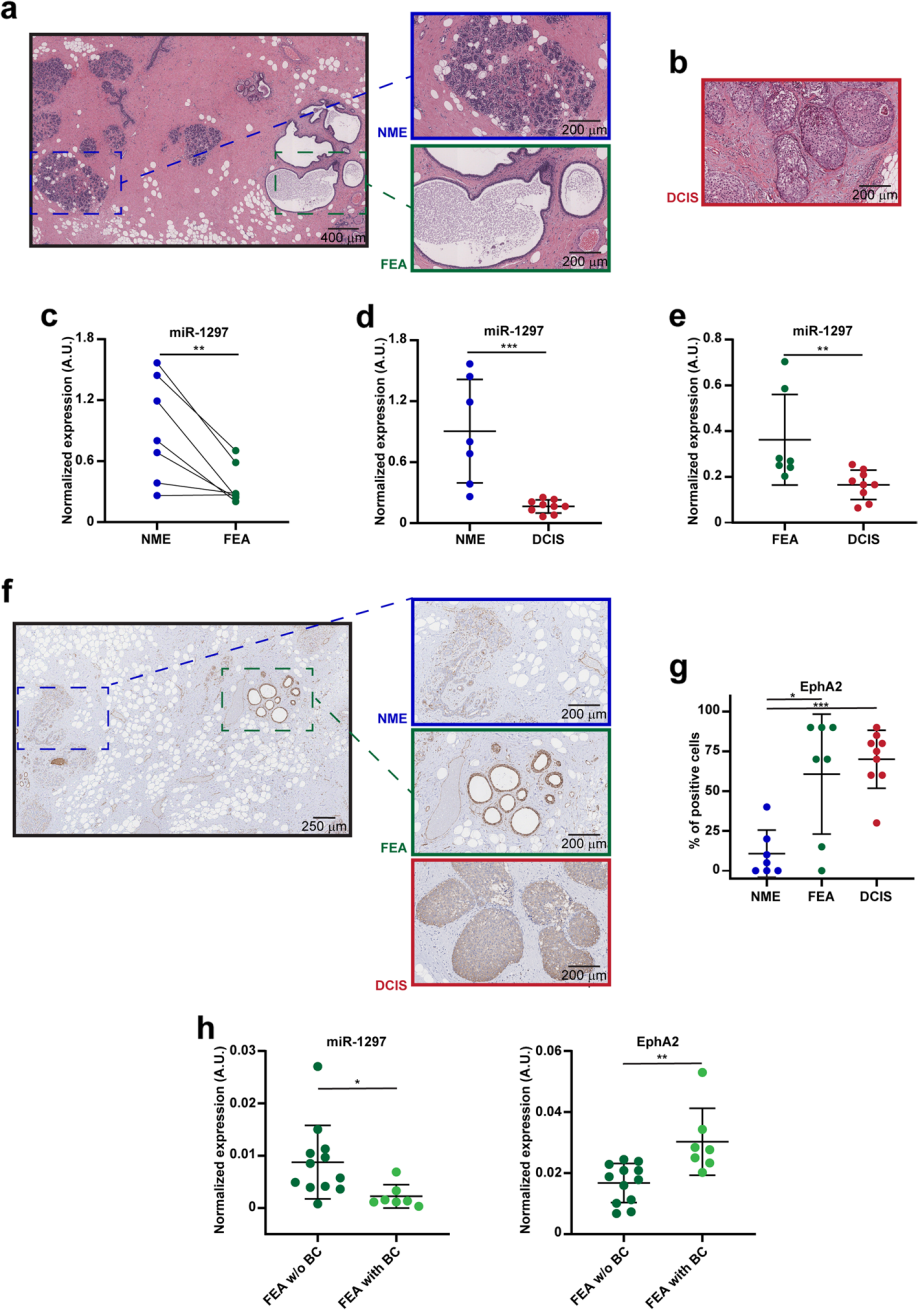
FEA is associated with miR-1297 downmodulation and EphA2 overexpression in an independent cohort of patients. **a**, On the left, a representative picture of H&E staining of a human breast tissue specimen is shown. From each specimen (n=7), areas of the normal mammary epithelium (NME, upper box in blue) and areas of flat epithelial atypia (FEA, lower box in green) were separately microdissected. **b,** Representative picture of H&E staining of a non-invasive ductal carcinoma in situ (DCIS). **c**, Graph reports data from RT-qPCR analysis of normalized miR-1297 expression (in arbitrary units, A.U.) in 7 specimens of FEA and the NME counterpart. A paired Wilcoxon test was used for statistical analysis and asterisks indicate the significative differences. ** p-value <0.01. **d, e,** Graphs report data from RT-qPCR analyses of normalized miR-1297 expression (in arbitrary units, A.U.) in specimens from patients with DCIS (n=9) compared with NME (on the left) and FEA (on the right) (n=7). **f,** Representative images of EphA2 immunohistochemical staining of human BC samples. In the boxes, areas of NME (upper box, in blue), FEA (middle box, in green) and DCIS (lower box, in red) that were separately microdissected from each specimen are shown. **g**, Dot plots showing the expression of EphA2 evaluated by immunohistochemistry in NME, FEA and DCIS, as indicated. Data represent the percentage of positive cells in each sample. **h**, Graphs show qRT-PCR analysis of normalized miR-1297 (left) and EphA2 expression (right) in arbitrary units (A.U.) in FEA samples from patients without (w/o, n=12) or with (n=7) BC. In **d**, **e, g** and** h**, an unpaired t-test was used for statistical analysis and asterisks were used to report the significative differences. * *p*-value <0.05; ** *p*-value <0.01; *** *p*-value <0.001.

To further corroborate these findings, we collected surgical specimens from a validation cohort of BC patients, including cases where FEA coexisted with areas of NME, as well as cases with DCIS. As conducted in the exploratory cohort, we performed microdissection on areas of interest within each specimen (Figure 2a, b) and quantitatively analyzed the expression of miR-1297 using qRT-PCR (Figure 2c-e). In line with the previous results, we found that FEA showed a marked reduction of miR-1297 levels compared with the adjacent normal tissue (Figure 2c), further decreasing in the DCIS regions (Figure 2d, e). Then, by analyzing EphA2 expression *via* immunohistochemistry (IHC) in the same context, we observed a significant upregulation in FEA and DCIS compared to the NME, indirectly confirming the molecular link with miR-1297 (Figure 2f, g). Next, we investigated whether the expression of our targets differed in FEA based on its association with BC in surgical specimens. To this end, we analyzed a set of patients in which FEA was either the final diagnosis (without BC) or found in association with BC. qRT-PCR analysis revealed a significant decrease in miR-1297 levels in FEA associated with BC compared to FEA as a final diagnosis, accompanied by increased EphA2 expression (Figure 2h). These findings further support the possibility that miR-1297 and EphA2 levels could influence the progression of FEA toward BC.

### miR-1297 expression inhibits the proliferation of BC cells *via* EphA2 targeting

Data collected so far suggested that miR-1297 expression is downregulated during the initial transformation of mammary epithelium towards FEA-DCIS. To delve deeper into the role of miR-1297 targeting EphA2, we moved to an *in vitro* setting.

**Fig 3.**
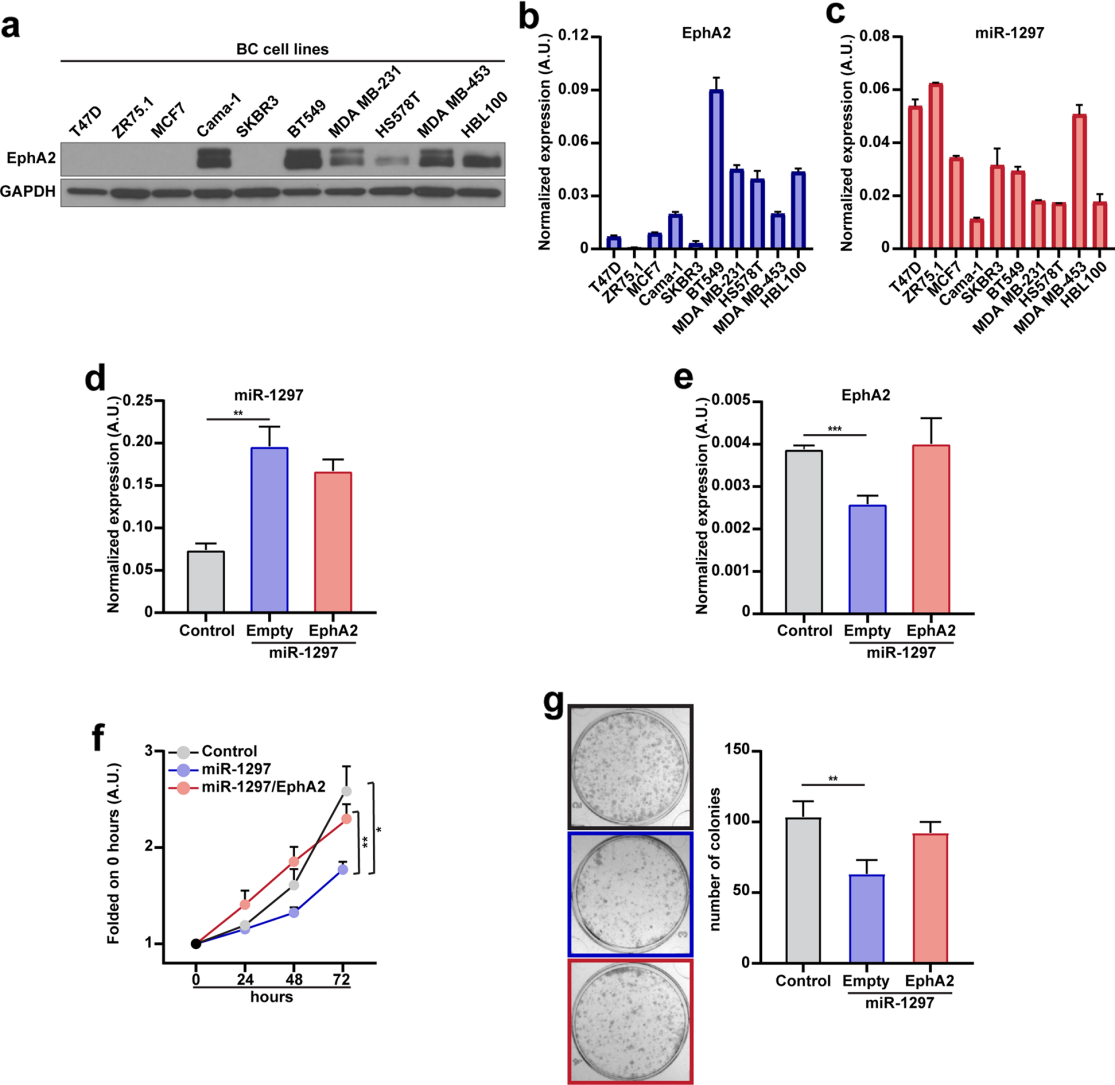
miR-1297 expression inhibits proliferation of BC cells *via* EphA2 targeting. **a**, Western blot analysis evaluating the expression of EphA2 in lysates from the indicated BC cell lines. GAPDH was used as the loading control. **b**, **c**, Histograms representing the normalized expression of EphA2 (**b**) and miR-1297 (**c**) by qRT-PCR in different BC cell lines, as indicated. **d**, **e**, Histograms report the normalized expression of miR-1297 (**d**) and EphA2 (**e**) evaluated by qRT-PCR analysis in control, miR-1297 transfected and miR-1297 transfected/EphA2 overexpressing BT549 cells. **f**, Graph reports the growth rate of control, miR-1297 transfected and miR-1297/EphA2 overexpressing BT549 cells over a period of 72 hours. Data are folded on the 0 h timepoint and represent the mean (±SD) of three independent experiments performed in triplicate. Two-way ANOVA was used to verify the statistical significance and asterisks indicate significant differences. **g**, Representative images (left) and graph (right) of colony formation assay of control, miR-1297 transfected and miR-1297 transfected/EphA2 overexpressing BT549 cells. Data represent the number of colonies from three independent experiments performed in duplicate. In **d**, **e,** and **g**, Significant differences were evaluated compared to the control, and an unpaired t-test was used for statistical analyses. * *p*-value <0.05; ** *p*-value <0.01; *** *p*-value <0.001

First, we investigated EphA2 expression across various BC cell lines (Figure 3a, b), revealing diverse protein and transcript levels. BT549 cell line exhibited robust EphA2 expression, as evidenced by both western blot and qRT-PCR analyses, coupled with low levels of miR-1297 (Figure 3a-c). Transfection of the miR-1297 construct into BT549 cells led to a substantial decrease in EphA2 levels compared to the control cells (Figure 3d, e). Additionally, miR-1297 transfection suppressed cell growth (Figure 3f) and the ability to form colonies (Figure 3g) compared to the BT549 control cells. Significantly, the anti-proliferative effect of miR-1297 specifically relied on EphA2 targeting, since the ectopic re-introduction of EphA2 in miR-1297 transfected cells restored the phenotype observed in the control (Figure 3d-g).

Altogether, our results supported that miR-1297 inhibits the proliferation of BC cells by repressing EphA2 expression.

### miR-1297 downregulation promotes the transformation of normal mammary epithelial cells

**Fig 4.**
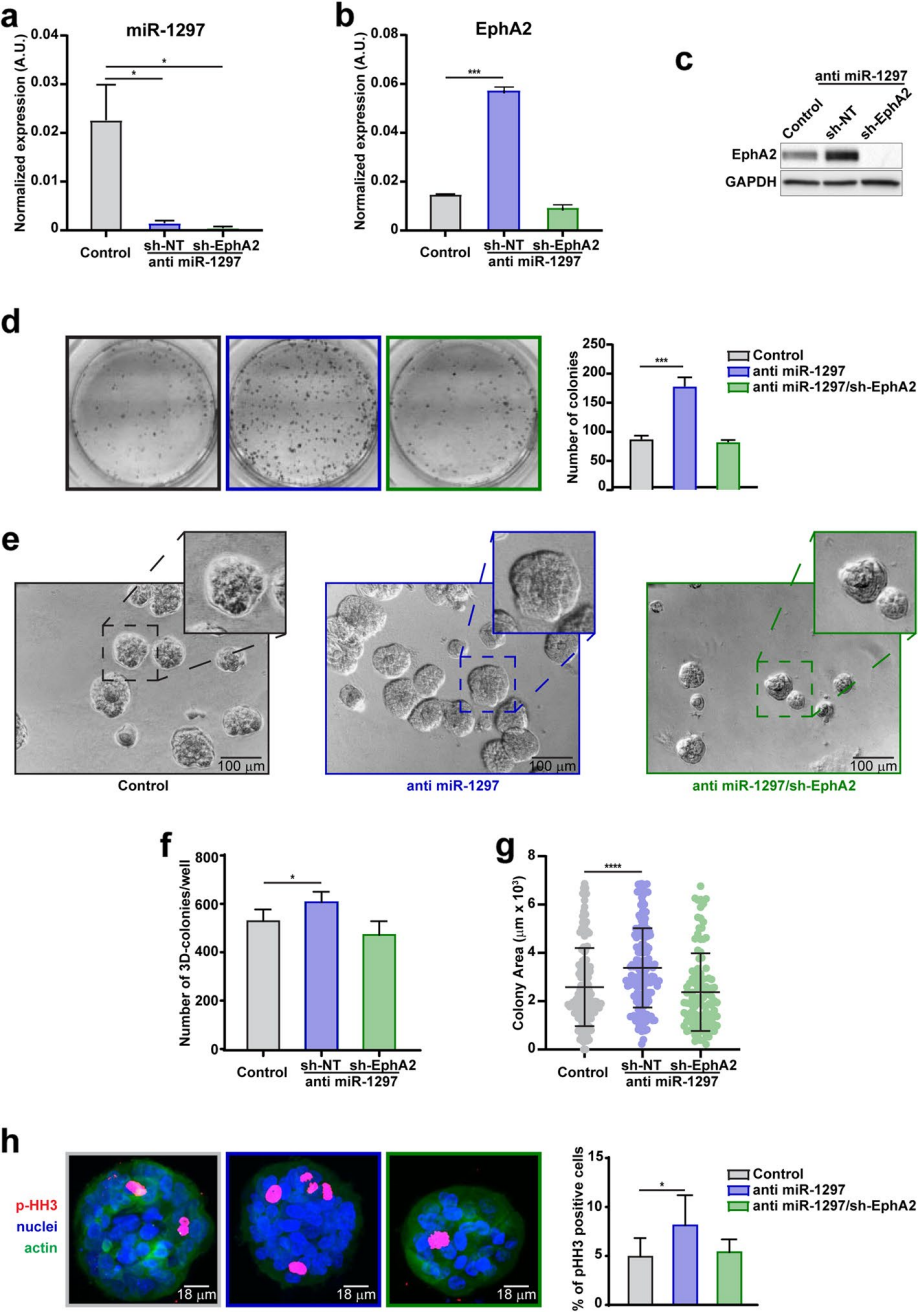
The downregulation of miR-1297 demonstrates transformative potential in normal mammary epithelial cells. **a**, **b**, Histograms report the normalized expression of miR-1297 (**a**) and EphA2 (**b**) by qRT-PCR analysis in control, anti miR-1297, and anti miR-1297/EphA2 silenced BPEC cells. **c**, Western blot analysis evaluating the expression of EphA2 in control, anti miR-1297, and anti -miR-1297/EphA2 silenced BPEC cells. GAPDH was used as the loading control. **d**, Representative images (left) and graph (right) of colony formation assay of control, anti-miR-1297, and anti-miR-1297/EphA2 silenced BPEC cells. Data represent the number of colonies from three independent experiments performed in duplicate. **e**, Representative contrast-phase images of control, anti miR-1297, and anti miR-1297/EphA2 silenced BPEC cells included in 3D Matrigel, and allowed to grow for 8 days. At the top right corner, insets show an enlargement of the 3D colonies to highlight differences in their size. **f,** Graph reports the colony number/well of the experiment described in **e**. **g,** Graph reports the colony area described in **e** measured using the Volocity software and expressed as μm^2^ x 10^3^. Each dot corresponds to one colony. **h**, Representative confocal images (right) and graph (right) of control, anti miR-1297, and anti miR-1297/EphA2 silenced BPEC cells of the experiment described in **e**. Mammary acini were immunostained for nuclei (TO-PRO-3, blue), actin (phalloidin, green), and pSer10 Histone H3 (p-HH3, red), and the graph reports the percentage of mitotic cells, as identified by the p-HH3 marker. Significant differences were evaluated in **a**, **b**, **d**, **f**,** g**, and **h**, compared to the control. Student t-test or Mann-Whitney test was used for statistical analysis, as appropriate. Asterisks indicate significant differences. * *p*-value <0.05; *** *p*-value <0.001; **** *p*-value <0.0001.

The data gathered from human samples suggested that the transition of NME towards FEA and DCIS is linked with a gradual decrease in miR-1297 expression (Figure 2c-e) alongside an increase in EphA2 levels (Figure 2g). Thus, we wondered whether miR-1297 loss could be functional in transforming normal epithelial cells *via* EphA2 targeting. To this aim, we manipulated normal breast primary epithelial cells, namely BPEC and HMEC cells, to achieve silencing of miR-1297 expression (Figure 4a and Supplementary Figure 2a), observing a subsequent increase in EphA2 levels (Figure 4b, c and Supplementary Figure 2b, c). Conversely, EphA2 overexpression in HMEC cells did not affect miR-1297 levels (Supplementary Figure 2d, e), ruling out the possibility of a reciprocal regulatory mechanism *via* target-RNA-directed microRNA degradation (TDMD) [33].

While testing their growth behavior in a two-dimensional context, miR-1297 silenced and EphA2 overexpressing cells more efficiently formed colonies than the control (Figure 4d and Supplementary Figure 2f, g). By contrast, the silencing of EphA2 in BPEC significantly reduced the number of colonies in anti miR-1297 cells (Figure 4a-d).

To better understand the effects of miR-1297 abrogation, we included BPEC and HMEC cells in a three-dimensional Matrigel culture. Upon examining the morphology, we noted a significant impact of miR-1297 silencing on the 3D formation of mammary acini. These structures appeared more disorganized, lacking the characteristic hollow lumen in HMEC cells (Supplementary Figure 2h). Moreover, the miR-1297 knockdown significantly increased the number and size of mammary acini, enhancing the mitotic rate compared to controls (Figure 4e-h, and Supplementary Figure 2i-k). Consistently, EphA2 overexpressing cells also formed larger mammary acini compared to the control (Supplementary Figure 2l). However, the EphA2 silencing reduced the number and size of anti miR-1297 mammary acini, restoring the proliferation rate observed in control cells (Figure 4e-h).

These findings support the role of miR-1297 downregulation in facilitating the acquisition of a transformed phenotype through the upregulation of EphA2 in normal mammary epithelial cells.

## Discussion

Over the last decades, numerous studies have elucidated the genetic basis of BC, and considerable efforts have been dedicated to correlating the clinical behavior of tumors with their underlying molecular mechanisms [34–36]. In this context, the dysregulation of microRNAs has been extensively involved in every step of BC progression, highlighting their potential usefulness as clinical markers for classifying tumor subtypes and guiding patient management [17, 37, 38]. Nevertheless, certain precursor entities such as FEA continue to evade clear understanding, presenting a persistent challenge from a clinical standpoint [4, 7, 9, 11].

To fill this gap, we performed the first comprehensive miRNAome study on FEA, specifically targeting the microRNAs that collectively characterize FEA alongside DCIS and distinguish them from the normal mammary epithelium (Figure 1a, b).

Among them, we focused on miR-1297 (Figure 1c), which has been involved in the tumorigenesis of different malignancies and is downmodulated in invasive BC [26–28]. Intriguingly, within our validation cohort, we observed a downregulation of miR-1297 in FEA compared to normal tissue, with a further decrease evident in DCIS samples (Figure 2c, e).

Notably, FEA without BC association retained higher miR-1297 levels and exhibited EphA2 downregulation compared to FEA associated with BC (Figure 2h), suggesting that miR-1297 dysregulation may be linked to the progressive transformation of mammary epithelium and the establishment of BC. Further studies involving larger cohorts of patients are needed to confirm these observations.

A limitation of our study is the lack of follow-up data on the progression of FEA with low miR-1297 levels toward malignancy. As a result, we are unable to determine whether miR-1297 downregulation can serve as a predictive biomarker for FEA progression. Clarifying these aspects will be highly relevant from a translational perspective. Indeed, the introduction of high-quality screening tests has led to the overdiagnosis of non-invasive breast lesions on biopsy. Nevertheless, no markers are currently available to identify biopsy-proven FEA associated with malignancy in the surgical specimen [1, 6, 7].

Our *in vitro* experiments showed that miR-1297 expression regulates both the proliferation and colony-forming capability of BC cells (Figure 3f, g), as well as the behavior of primary mammary cell lines (Figure 4d and Supplementary Figure 2f). Exploiting a model of mammary acini in a three-dimensional Matrigel, we noted that downregulation of miR-1297 resulted in increased proliferation and disrupted the normal conformation of acini (Figure 4e-h and Supplementary Figure 2h-l). These findings are particularly significant considering the histological definition of those breast precursor lesions, which relies on criteria such as cell hyperplasia and architectural features [8].

On a mechanistic level, we showed that miR-1297 exerts its role by directly targeting EphA2 (Figure 1d-f, and 3e-g, and 4d-h) that, consistently, is differently expressed in the progression from NME towards FEA and DCIS (Figure 2f, g). Literature evidence reports that EphA2 levels are linked with poor prognosis, increased risk of metastatic dissemination and reduced survival in BC patients [30, 39, 40]. Our work suggests the involvement of EphA2 in the earliest stage of mammary tumorigenesis and elucidates the molecular mechanism underlying its overexpression. This discovery holds direct clinical relevance, as EphA2 is a therapeutic target, and selective inhibitors and antibodies are currently being tested in several clinical trials [31, 41, 42].

In summary, our findings shed light on the molecular pathways involved in transitioning from benign to pre-malignant stages in BC development, highlighting the potential role of miR-1297 and EphA2 as therapeutic targets or biomarkers for early detection and intervention.

## Materials and methods

### Human specimen collection, study approval and histological analysis

Formalin-fixed and paraffin-embedded (FFPE) tissue specimens were retrospectively collected from patients with or without primary BC upon signing a written informed consent, in accordance with recognized ethical guidelines (Helsinki Declaration) and followed by approval of The University of Rome “Sapienza”, Sant’ Andrea Hospital (Rome, Italy). The areas of interest were selected by two pathologists jointly and microcaptured using laser microdissection technology (LMD) as previously described [22]. Three cohorts were analyzed: an exploratory set (including n= 59 NME, 38 FEA, and 42 DCIS) and two validation cohorts. The first validation cohort consisted of n=7 cases of NME and n=7 adjacent FEA from the same patient and n=9 cases of DCIS collected from BC patients. The second validation cohort included n=12 cases of FEA not associated with BC and n=7 cases of FEA associated with BC. For each lesion, 10 microdissections of approximately 8 μm thickness were performed as part of the sampling process.

For histological analysis, FFPE tissue blocks were sectioned at a thickness of 2 μm, deparaffinized using PT Link (Agilent, USA), and then rehydrated. The antigen retrieval was obtained at a high pH. The peroxidase blocking solution (Agilent, USA) was used to inhibit endogenous peroxidase for 10 minutes. Tissue slices were coated with Dako EnVisionTM FLEX /HRP (EnVisionTM FLEX; Agilent, USA) for 30 minutes at room temperature after being immunostained for 1 hour at room temperature using anti-EphA2 Polyclonal Goat IgG (1:100, Cat. #AF3035, R&D System, USA). The DAB detection kit (Agilent, USA) was used to produce the signal, and Mayer's Hematoxylin (Agilent, USA) was used to counterstain the sections. After washing and dehydrating the slides with xylene and increasing alcohol, the slides were mounted using Eukitt (Sigma-Aldrich, USA).

### RNA extraction and hybridization to nCounter tagset

The NanoString nCounter Human miR Expression Assay Kit (Nanostring Technologies, USA) was used to profile more than 800 human miRNAs in NME, FEA and DCIS from the explorative cohort. RNA was extracted with High Pure FFPE RNA Isolation Kit (Roche Diagnostics, Switzerland) according to the manufacturer's recommendations and quantified using fluorimetry with Qubit RNA HS Assay Kit (ThermoFisher Scientific, USA). A total of 100 ng RNA was used as input for nCounter miRNA sample preparation reactions. Data collection was carried out on the nCounter Digital Analyzer (NanoString Technologies, USA) following the manufacturer’s instructions and as previously described [43], counting individual fluorescent barcodes and quantifying target RNA molecules present in each sample. For each assay, a high density (600 fields of view) was performed. Detailed information is reported in Supplementary Data.

### Cell lines and transfection

BPE-3 cells (hereafter BPEC) were purchased from LTCC (Live Tissue Culture Service Center-LTCC@med.miami.edu) and grown in BMI-P medium (LTCC), supplemented with cholera toxin 100 ng/mL (Sigma Aldrich, USA), as previously reported [22] and following the manufacturer's instructions. All other cell lines were obtained from the ATCC, maintained in culture following the ATCC indications, and grown in a controlled environment of 37°C and 5% CO2. For transient transfection, pre-miR-1297 precursor (Cat. PM13747), pre-miR precursor Negative Control #1 (Cat. AM17110), and anti miR-1297 (Cat, AM13747) were purchased from ThermoFisher Scientific, USA. EphA2-YFP was a gift from Kalina Hristova [44] (plasmid #108852, Addgene). EphA2 silencing was obtained by combining TRCN0000231648 and TRCN0000006403 (Millipore Sigma, USA). Lipofectamine 2000 (ThermoFisher Scientific, USA) transfection system was used following the manufacturer’s instructions in BT549, BPEC and HMEC breast cell lines.

### Luciferase activity assays

The predicted miR-1297-binding site of EPHA2 3’UTR was amplified by PCR using specific primers. PCR products were digested and cloned into the pMir target vector. The mutant of EPHA2 3’UTR was generated using QuikChange II XL Site-Directed Mutagenesis Kit (Agilent, USA), according to the manufacturer’s protocol and as described [20]. MCF7 breast tumor cells were transfected with either pMir- 3'UTR EPHA2 WT or mutated plasmid together with 100 ng of β-GAL vector and 50 pmoles of pre-miR-1297 or pre-miR precursor Negative Control #1 (Thermo Fisher Scientific, USA); a control plasmid expressing Renilla luciferase was co-transfected at the same time. Transfections were carried out using Lipofectamine 2000 (Thermo Fisher Scientific, USA). After 48 hours from transfection, luciferase activity was measured using the Luciferase Reporter Assay (Promega, USA). Each transfection was repeated in three independent experiments. Transfection efficiency was corrected to β-GAL expression in all cases.

### Colony assay and growth curve analysis

To evaluate the proliferation rate, BT549 cells were transfected with control, miR-1297 and miR-1297/EphA2 as indicated and, after 5 hours, were counted and seeded into 96-well plates (1000 cells/well) and maintained for 72 hours. Cell growth was monitored at the indicated time points using RealTime-Glo^TM^ MT Cell Viability assay (Cat. #G9713, Promega, USA). Colony formation assay was performed in BT549, BPEC and HMEC cells transfected as indicated and seeded into 6-well plates (1-2x10^3^ cells/well) and maintained for 7-14 days, depending on the cell type. Colonies were then fixed, stained with crystal violet solution (0.5 mg/ml in 20% methanol) and counted manually.

### Three-dimensional mammary epithelial cell cultures

Three-dimensional (3D) cell culture was performed essentially as previously described [22]. HMEC and BPEC cells (5-8x10^3^ cells) were seeded in an 8-well Labtek chamber slide (Becton Dickinson, USA), embedded as single cells in Cultrex® Growth Factor Reduced Basement Membrane Extract (GFR-BME, 2%) (Trevigen, USA), mixed with the appropriate medium and layered on the top of a bottom layer of polymerized GFR-BME (8.5mg/ml) (Trevigen). For 8 days, the embedded cells were cultured at 37°C and fresh medium was added every 3 days. Using Volocity® (PerkinElmer) software, the number of acini was counted at the end of the experiment and photos were gathered to determine colony number and areas.

Morphological evaluations were performed by immunofluorescence analysis, essentially as described previously [22, 45]. Briefly, mammary acini grown in 3D culture were fixed in PFA at 4°C ON, incubated with primary antibodies ON at 4°C, followed by 1h at RT with secondary antibody. Nuclei were counterstained using TO-PRO-3 (Invitrogen, USA) for 30 min at RT. Samples were analyzed using a confocal laser-scanning microscope (TSP8, Leica, Germany) interfaced with a Leica fluorescent microscope.

Primary antibodies used were pSer10 Histone H3 (Cat. #06-570, Millipore, USA) and Keratin 14 (Cat. #905301, Biolegend, USA). Phalloidin (Alexa Fluor 546, #A22283, Invitrogen, USA) was used to stain F-Actin.

### qRT-PCR and Western blot analyses

As described above, RNA was isolated from microdissected NME, FEA, and DCIS with High Pure FFPE RNA Isolation Kit (Roche Diagnostics, Switzerland), as described above. RNA from mammary cells was extracted using TRIzol reagent (Invitrogen, USA). RNA was retro-transcribed using the TaqMan® Advanced miRNA cDNA Synthesis Kit (Thermofisher Scientific, USA) for microRNAs, and the Premix Ex Taq™ Probe qPCR (Takara Bio, Japan) for genes assessment, in accordance with the manufacturer’s instructions. qRT-PCR analyses were performed with TaqMan™ Fast Advanced Master Mix (Thermofisher Scientific, USA), using the following probes purchased by Thermofisher Scientific, USA: miR-1297 (479392_mir Cat. A25576), miR-let-7a-5p (478575_mir_ Cat. A25576), Epha2 (Hs01072272_m1), and beta-Actin (Hs01060665_g1). The data was normalized using miR-let-7a-5p and beta-Actin, and relative expression was determined using the 2−ΔΔCT method.

Western Blot analysis was performed essentially as previously described [22, 45]. Cell lysates were prepared using ice-cold RIPA buffer (Sigma Aldrich, USA) and Complete inhibitor (Roche, Switzerland). Protein concentrations were determined using Bradford’s method 595nm (Bio-Rad protein assay kit, USA). Protein lysates were separated in 4-20% SDS-PAGE (Criterion TGX Precast Gel, Bio-Rad, USA) and transferred to nitrocellulose membranes (GE Healthcare, USA). Membranes were blocked with 5% non-fat dried milk (NFDM) in TBS-0.1% Tween-20 and incubated at 4°C overnight (ON) with primary antibodies. Primary antibodies used were: EphA2 (Cat. sc-398832, Santa Cruz Biotechnology, USA), Actin (Cat. sc-47778, Santa Cruz Biotechnology, USA), and GAPDH (Cat. 5174, Cell Signaling Technology, USA). Following incubation with primary antibody, membranes were washed in TBS-0.1% Tween 20 and incubated 1 hour (h) at room temperature (RT) with the horseradish peroxidase-conjugated secondary antibodies (GE Healthcare, USA) for ECL detection (Clarity Western ECL Substrate, Bio-Rad, USA).

### Statistical analyses and reproducibility

Statistical significance, averages, median, and SD were determined using GraphPad PRISM software (version 6.01), using the most appropriate test specified in each figure. Significance is indicated by a *p* < 0.05.

## Supplementary Information

Supplementary Material 1.

Supplementary Material 2.

Supplementary Material 3.

Supplementary Material 4.

## Data Availability

Raw data supporting this study’s findings have been obtained by NCBI Gene Expression Omnibus (GEO; https://www.ncbi.nlm.nih.gov/geo/) and are under accession number GSE41970. All data reported in this manuscript are available upon request.
