# Supplementary material for: miR-1297 is frequently downmodulated in flat epithelial atypia of the breast and promotes mammary neoplastic transformation via EphrinA2 regulation
Source: J Exp Clin Cancer Res. 2025 Mar 14;44:96. doi: 10.1186/s13046-025-03354-2 (PMC11908103; doi:10.1186/s13046-025-03354-2)
Supplement: Supplementary file 1 — Supplementary Material 1. [file 13046_2025_3354_MOESM1_ESM.docx]

**Supplementary Materials for**

**miR-1297 is frequently downmodulated in flat epithelial atypia of the breast and promotes mammary neoplastic transformation *via* EphrinA2 regulation.**

Giorgia Scafetta^1^*****°, Gian Luca Rampioni Vinciguerra^1^*****, Simona Giglio^1^, Omar Faruq^1^, Roberto Cirombella^1^, Ilenia Segatto^2^, Francesca Citron^2^, Maria Chiara Mattevi^2^, Elisabetta Di Renzi^1^, Luciano Cascione^3^, Pierluigi Gasparini^4^, Barbara Belletti^2^, Gustavo Baldassarre^2^, Andrea Sacconi^5^, Giovanni Blandino^6^, and Andrea Vecchione^1^.

^1^Department of Clinical and Molecular Medicine, Faculty of Medicine and Psychology, Sant'Andrea Hospital, University of Rome "Sapienza", Rome, 00189, Italy.

^2^Unit of Molecular Oncology, Centro di Riferimento Oncologico di Aviano (CRO), IRCCS, National Cancer Institute, 33081, Aviano, Italy.

^3^Institute of Oncology Research, Faculty of Biomedical Sciences, USI, Bellinzona, Switzerland.

^4^Department of Cancer Biology and Genetics and Comprehensive Cancer Center, The Ohio State University, Columbus OH, 43210, USA.

^5^Clinical Trial Center, Biostatistics and Bioinformatics Unit, IRCCS Regina Elena National Cancer Institute, Rome, Italy.

^6^Translational Oncology Research Unit, IRCCS Regina Elena National Cancer Institute, Rome, Italy.

*****These authors contributed equally to this work.

°Present address: Translational Oncology Research Unit, IRCCS Regina Elena National Cancer Institute, Rome, Italy.

Correspondence should be addressed to: Andrea Vecchione (andrea.vecchione@uniroma1.it) or Gian Luca Rampioni Vinciguerra (gianluca.rampionivinciguerra@uniroma1.it)

**This PDF file includes:**

Supplementary Figures 1, 2

Legend of Supplementary Tables 1-3

Supplementary Methods


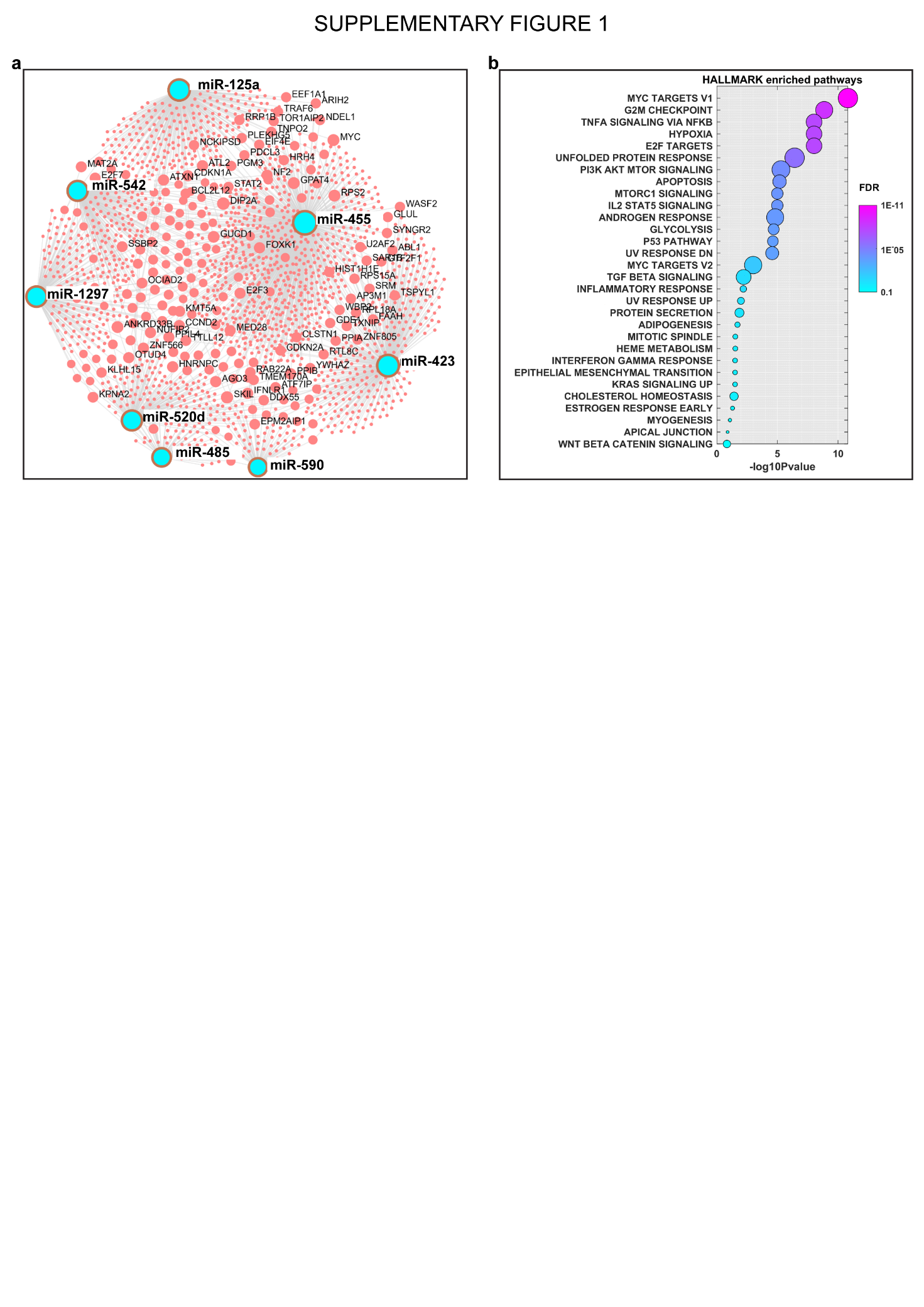


**Supplementary Figure 1. a**, miRNA-mRNA network obtained from downregulated miRNAs in FEA and DCIS compared to NME using OmicsNet (https://www.omicsnet.ca/). Experimentally validated miRNA-mRNA interactions were assessed from miRTarBase (v8.0). This procedure highlighted a subnetwork comprising 8 miRNAs out of the 33 downregulated miRNAs identified. **b**, Bubble plot showing the Enriched HALLMARK pathways obtained from validated targets (miRTarBase v8.0) of 8 miRNAs included in the miRNA-mRNA network. The enrichment was evaluated using ShinyGO 0.80 (http://bioinformatics.sdstate.edu/go/).

**
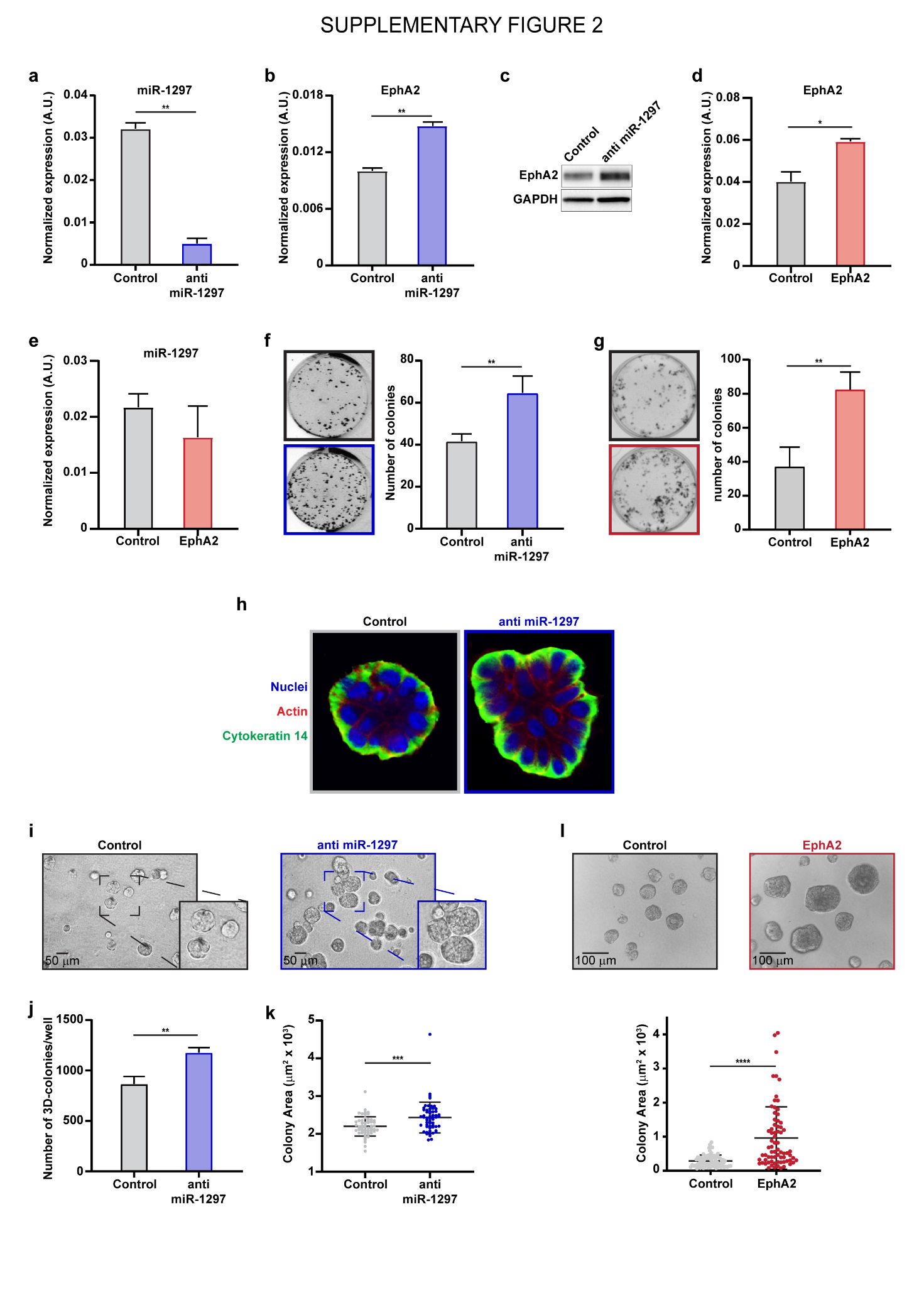
Supplementary Figure 2. a**, **b**, Histograms report the normalized expression in arbitrary units (A.U.) of miR-1297 (**a**) and EphA2 (**b**) evaluated by qRT-PCR analysis in control and anti-miR-1297 HMEC cells. **c**, Western blot analysis evaluating the expression of EphA2 in control and anti-miR-1297 HMEC cells. GAPDH was used as loading control. **d**, **e**, Histograms report the normalized expression in arbitrary units (A.U.) of EphA2 (**d**) and miR-1297 (**e**) evaluated by qRT-PCR in control and EphA2-overexpressing HMEC cells. **f**, **g**, Representative images (left) and graph (right) of colony formation assay of control and anti-miR-1297 (**f**), and EphA2-overexpressing (**g**) HMEC cells. Data represent the number of colonies from three independent experiments performed in duplicate. **h**, Representative confocal images of mammary acini from control and anti-miR-1297 HMEC cells, included in 3D Matrigel and allowed to grow for 10 days. Mammary acini were immunostained for nuclei (TO-PRO-3, blue), actin (phalloidin, red), and Cytokeratin 14 (green). **i**, Representative contrast-phase images of control and anti–miR-1297 HMEC cells from the experiment described in **h**. At the bottom right corner, insets showing an enlargement of the 3D-colonies to highlight differences in their size. **j**, Graph reports the colony number/well of the experiment described in **h**. **k,** Graph reports the colony area of the experiment described in **h** measured using the Volocity software and expressed as μm^2^ x 10^3^. Each dot corresponds to one colony. **l**, Representative contrast-phase images (top) and graph (bottom) of colony formation assay of control and EphA2-overexpressing HMEC cells, included in 3D Matrigel and allowed to grow for 10 days. Graph reports the colony area measured using the Volocity software and expressed as μm^2^ x 10^3^. Each dot corresponds to one colony.

In **a**, **b**, **d**, **f**, **g, j, k** and **l**, Significative differences were evaluated compared to the control. Student t-test or Mann-Whitney test was used for statistical analysis, as appropriate. Asterisks indicate significant differences. ** p-value <0.01; *** p-value <0.001.

**Legend of Supplementary Table 1.** Table reports the results of the DEseq analysis of 89 miRNAs up and down regulated. Average counts of the two subgroups analyzed; p-value and adjusted p-value for multiple comparisons; Wilcoxon test values are reported.

**Legend of Supplementary Table 2.** Table reports the lists of validated target genes of the miRNAs signature as reported by miRTarBase v8.0.

**Legend of Supplementary Table 3.** The table reports enriched HALLMARK pathways obtained from validated targets (miRTarBase v8.0) of 8 miRNAs included in the miRNA-mRNA network, as listed in Supplementary Table 2. The enrichment was evaluated using ShinyGO 0.80 (<http://bioinformatics.sdstate.edu/go/>).

**SUPPLEMENTARY MATERIALS AND METHODS**

**NanoString nCounter and Data analysis**

The expression levels of 800 miRNAs were assessed in RNAs extracted from patients using the NanoString nCounter Human miR Expression Assay Kit (NanoString) 100ng of total RNA were used as input for nCounter miRNA sample preparation reactions. All sample preparation was performed according to the manufacturer’s instructions (NanoString Technologies, USA). Preparation of small RNA samples involves the ligation of a specific DNA tag onto the 3′ end of each mature miR. These tags are designed to normalize the Tms (melting temperatures) of the miRs as well as provide a unique identification for each miR species in the samples. The tagging is accomplished in a multiplexed ligation reaction using reverse complementary bridge oligonucleotides to direct the ligation of each miR to its designated tag. After the ligation reaction, excess tags and bridges are removed, and the resulting material is hybridized with a panel of miR tag specific nCounter capture and barcoded reporter probes. Hybridization reactions were performed according to the manufacturer’s instructions with 5 μL fivefold diluted sample preparation reaction. All hybridization reactions were incubated at 64 °C for a minimum of 18 h. Hybridized probes were purified using the nCounter Prep Station (NanoString Technologies) following the manufacturer’s instructions to remove excess capture and reporter probes and immobilize transcript-specific ternary complexes on a streptavidin coated cartridge. The nCounter Digital Analyzer collected the data by taking images of immobilized fluorescent reporters in the sample cartridge with a CCD camera through a microscope objective lens. For each cartridge, a highdensity scan encompassing 325 fields of view was performed. Images were processed internally into a digital format (RCC files). NanoString raw data were analyzed with nSolver, a software provided by NanoString Technologies. Negative controls were used to perform background subtraction. Positive controls were used to perform technical normalization to adjust any lane-by-lane variability due to differences in hybridization, purification or binding. A preliminary technical normalization was performed on the positive control count values. The rows of the count table represent miRNAs, while the columns represent different conditions. Initially, each condition was normalized by dividing it by a library size factor. This size factor was calculated as the median ratio of each feature over the geometric mean of the feature across all conditions. MiRNAs with average counts less than 30 were excluded from further analysis. The normalized counts were then transformed using a log2 function. For each condition, a coefficient of variation was calculated, and miRNAs with a coefficient of variation < 0.1 were selected for subsequent analyses.

Deregulated miRNAs in both tumoral vs. normal samples and FEA vs. normal samples were identified using a Negative Binomial test, and the resulting p-values were adjusted for multiple comparisons using the Benjamini and Hochberg procedure. Unsupervised hierarchical clustering with Euclidean distance and principal component analysis (PCA) were performed on the normalized counts. From the deregulated miRNAs, those exhibiting the most negative modulation were selected to construct a miRNA-mRNA network using OmicsNet (https://www.omicsnet.ca/). Experimentally validated miRNA-mRNA interactions were obtained from miRTarBase (v8.0). This procedure highlighted a subnetwork comprising 8 miRNAs out of the 33 downregulated miRNAs previously identified.The prediction of targets for the 8 selected miRNAs was assessed using TargetScanHuman, release 8.0 (https://www.targetscan.org/vert_80/). All the analyses were conducted by Matlab R2023b.
